# Comparative Effectiveness of Different Combinations of Treatment Interventions in Patients with Stroke at the Convalescence Stage Based on the Markov Decision Process

**DOI:** 10.1155/2020/8961341

**Published:** 2020-05-12

**Authors:** Yejing Shen, Mengyun Hu, Qianglong Chen, Yanyang Zhang, Junying Liang, Tingting Lu, Qinqin Ma, Ruijie Ma

**Affiliations:** ^1^The Third Clinical Medical College of Zhejiang Chinese Medical University, Hangzhou 310053, China; ^2^Acupuncture Department, Lishui Hospital of Traditional Chinese Medicine, Lishui 323000, China; ^3^Hospital of Zhejiang People's Armed Police, Hangzhou 310053, China; ^4^College of Computer Science and Technology, Zhejiang University, Hangzhou 310053, China; ^5^Quzhou Kecheng People's Hospital, Quzhou 324000, China; ^6^Zhoushan Dinghai Guanghua Hospital, Zhoushan 316000, China; ^7^Ningbo Haishu Rehabilitation Hospital, Ningbo 315000, China; ^8^The First Affiliated Hospital of Wenzhou Medical University, Wenzhou 325000, China; ^9^The Third Affiliated Hospital of Zhejiang Chinese Medical University, Hangzhou 310053, China

## Abstract

**Objective:**

The objective of this study was to compare the effectiveness of different combinations of interventions in patients with stroke at the convalescence stage based on the electronic health records (EHRs) by using the Markov decision process (MDP) theory and explore the feasibility of the Markov model in the real-world study (RWS).

**Methods:**

Screening was conducted for patients with stroke at the convalescence stage who were admitted to the Third Affiliated Hospital of Zhejiang Chinese Medical University from January 2012 to January 2017 based on the EHRs. The relevant clinical data were extracted, and the appropriate conversion was made (state-action-reward) according to the Markov model. The transformed data were analysed and solved by the MDP to obtain the best interventions for patients with various stroke recovery periods.

**Results:**

926 patients with stroke at the convalescence stage were initially selected. And according to the inclusion exclusion criteria, 854 patients were screened. Through the MDP, we obtained the following results: (1)when the patients with stroke at the convalescence stage have a medical history, but no complications, and mild neurological impairment, ≥66-year- and 18–45-year-old patients are advised to choose acupuncture treatment. 46–65-year-old patients are advised to choose rehabilitation treatment. When patients with moderate to severe neurological impairment, patients are advised to choose rehabilitation, Chinese herbal decoction, and acupuncture combined therapy. (2) Without complications or medical history, patients who are ≥ 66 years old are recommended to choose rehabilitation treatment when the nerve function impairment is mild; rehabilitation and acupuncture treatment are recommended when moderate and severe injuries are caused. (3) The combination of rehabilitation, Chinese herbal decoction, and acupuncture treatment is recommended for patients with phlegm and blood stasis. Acupuncture treatment is recommended for patients with mild impairment of nerve function in qi deficiency and blood stasis type. Rehabilitation, Chinese herbal decoction, and acupuncture treatment are recommended for moderate-severe injuries.

**Conclusions:**

The MDP makes it possible to study the effectiveness of various treatment methods in stroke patients who are at the convalescence stage. Further exploratory studies using MDP theory in other areas in which complex interventions are common would be worthwhile.

## 1. Introduction

Comparative effectiveness research (CER) belongs to the RWS and is also one of the clinically effective studies, also known as a “patient-centered outcome study.” CER combines such findings as the patients' basic characteristics, economic conditions, and personal preferences and then compares the advantages and disadvantages of various current medical measures to determine the best treatment plan for individuals [1, 2]. CER improves comprehensive medical services, such as prevention, diagnosis, and treatment of certain diseases and conditions [3]. CER is divided into observational research and nonobservational research. The core of observational research is to collect large-scale data sets, including patient signs, costs, and curative effects, from health management databases and EHRs. And the data are subsequently mined, calculated, analysed, compared, and quantified by researchers.

The MDP refers to the decision-maker's observation of the stochastic dynamic system with Markovity, periodically or continuously, and making decisions sequentially [4]. According to the state observed at each moment, an action is selected from the set of available actions, and the next state (future) of the system is decided by the environment and created by the observed data set. The probability of the transferred state has Markov property. The decision-maker makes new decisions based on the newly observed state and repeats this action accordingly. Because of its advantages in optimizing medical resources and selecting methods of diagnosis and treatments, MDP theory has played an important role in decision-making in the context of medical equipment management, hospital management, operation time, and adjustment of treatment strategies [5–8].

Stroke is one of the four major chronic diseases which include wind, consumptive disease, bloat, and dysphagia of traditional Chinese medicine (TCM) [9]. “Stroke” in modern medicine is a general term in TCM for acute cerebrovascular diseases. According to the statistics of the World Health Organization [10], in the 20th century, stroke was the second highest cause of death. At present, the annual incidence rate is 120/100,000 to 180/100,000, and the mortality rate is 60/100,000 to 120/100,000. Stroke has the characteristics of high incidence, high mortality, high disability rate, and many complications. Stroke threatens the health of many people and brings a heavy burden to patients, their families, and society [11, 12], which has become a global public health problem [13]. In 2015, the China Stroke Association first reported [14] that stroke was the main cause of death among Chinese residents in the *Chinese Stroke Epidemic Report*. TCM holds that the underlying pathogenesis is a dysfunction of yin and yang and a disordered flow of qi and blood, which then invade the brain [15]; therefore, stroke is inextricably linked with wind, fire, sputum, stagnation, qi, and deficiency.

Currently, patients with stroke at the convalescence stage are always treated with a combination of Chinese and modern medicine. At present, studies [16] have shown that the use of TCM or acupuncture has a significant effect on stroke patients at the convalescence stage. However, the question of how to comprehensively evaluate the combination of traditional Chinese and Western medicine treatment for stroke is a major clinical issue today. The main goal of this study is how to improve treatment efficacy and choose the optimal treatment to patients.

To address these issues, the author collected the hospitalized EHRs of patients with stroke at the convalescence stage who were admitted to the Third Affiliated Hospital of Zhejiang Chinese Medical University from January 2012 to January 2017. The report is as follows.

## 2. Methods

### 2.1. Establishment of the Data Set

#### 2.1.1. Data Collection

The hospital information staff and clinical professionals were responsible for the data collection from the EHRs, and 926 patients were initially selected. The inclusion criteria were a primary diagnosis of atherosclerotic cerebral infarction, cerebral embolism, or cerebral haemorrhage and between 30 and 180 days after the onset of stroke. Patients who had the occurrence of cerebrovascular accident before or experienced limb motor dysfunction due to other reasons were excluded. Patients were eliminated from the study if other interventions (such as surgery, endovascular intervention, and arteriovenous thrombolysis) were performed during hospitalization.

According to the inclusion and exclusion criteria, 854 patients were screened. The extracted information on the patients included the basic hospitalization information, diagnosis, medical information, physical examination, and other modules.

The study has passed the ethical review of the Ethics Committee (ZSLL-KY-2015-044), and we respected and protected the privacy of patients. All of the data were collected with an information acquisition form that captured the general information of the patient, TCM and Western medicine diagnosis, all applied treatments with course detail, physical examinations, and so on.

#### 2.1.2. Data Standardization

The standardization of Western medicine diseases was carried out according to the International Classification of Diseases Code ICD-10. TCM diagnostic syndrome information was standardized according to *Traditional Chinese Medicine Diagnostics* [17].

#### 2.1.3. Data Clean-Up and Set Establishment

The data in the EHRs were extensive; thus, the occurrence of abnormal data could not be avoided. Abnormal data could lead to deviations in the analytic results and affect the authenticity of real-world evidence. Therefore, in this study, abnormal values were eliminated. Based on the research purpose, clinical professionals created analytical data sets. Through the verification of the data, the missing data were found and filled in.

### 2.2. MDP Analysis

**Figure 1:**
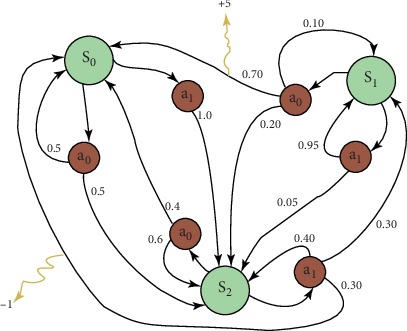
Schematic diagram of an MDP.

Figure 1 is a schematic diagram of an MDP with three states and two actions (from http://en.wikipedia.org/wiki/Markov decision process). In this study, S_0_, S_1_, and S_2_ correspond to patient states with different combinations of variables. Then, *a*_0_ and *a*_1_ represent different treatment combinations (actions). These actions have a certain probability in patients with different states, according to probability transfer and average benefits to calculate which action is the optimal solution. The data of this study belonged to the discrete distribution and could be calculated and solved by using a finite MDP.

**Figure 2:**
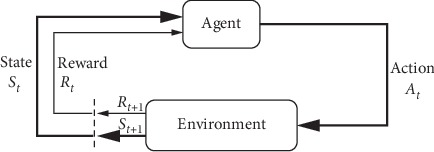
The agent-environment interaction in an MDP.

In a finite MDP, the sets of states, actions, and rewards (S, A, and R) all have a finite number of elements. In this case, the random variables *R*_t_ and *S*_t_ have well defined discrete probability distributions dependent only on the preceding state and action [18] (see Figure 2).

#### 2.2.1. Data Extraction and Conversion

To determine the key characteristics for describing the condition of patients with stroke at the convalescence stage and the criterion to be optimized by using MDP theory, a panel was formed that included scholars, professors, computer experts, physicians of Western medicine, TCM practitioners, and graduate students.

Based on the basic characteristics of patients with stroke at the convalescence stage, six aspects were established (see Additional file 1: Appendix 1): including (*i*_*1*_) age, (*i*_*2*_) medical history, such as diabetes, hypertension, coronary heart disease, abnormal blood liquid level, or auricular fibrillation, (*i*_*3*_) complications, such as pulmonary infection, urinary tract infection, or deep vein thrombosis, (*i*_*4*_) Western medicine diagnosis, (*i*_*5*_) syndrome differentiation, such as wind-fired disturbance type, phlegm-blocking type, yin-deficiency type, qi deficiency and blood stasis type, and phlegm-heat type, and (*i*_*6*_) neurological function impairment scores. A score was used to describe the level of neurological function defect. The total scores were in the range of 0–29. The scores were divided into five grades, as follows: level 1 (0–2), level 2 (3–5), level 3 (6–12), level 4 (13–19), and level 5 (20–29) (see Additional file 1: Appendix 2). For the patient with an outcome of death, the default score was 29. Connected with clinical practice, level 1 and 2 represent that the severity of neurological deficits is mild, level 3 is moderate damage, and level 4 and 5 are severe injuries.

Action is known as treatment. All patients were given Western medicine for secondary prevention. Thus, the treatment methods were divided into the following three circumstances (actions) *a*_*1*_–*a*_*3*_ (see Additional file 1: Appendix 1): (*a*_*1*_) whether to use rehabilitation therapy; (*a*_*2*_) whether to use Chinese herbal decoction; and (*a*_*3*_) whether to use acupuncture treatment. In this study, treatment strategies were carried out at the request of the physician in charge of the patient under the same theory of TCM [19]. A patient who treated by a rehabilitation specialist was considered to receive undergone rehabilitation treatment; otherwise, the one was not considered to use rehabilitation. The clinical treatment of Chinese herbal decoction and acupuncture must be consistent with TCM syndrome differentiation.

This reward refers to the value of the differential between the scores for neurological function impairment before and after treatment. According to the expert's advice, the total reward values became the criteria to be optimized. If the value was 0, the patient's condition was no change; if the improvement value was >0, the patient's condition could be improved with the corresponding treatment plan. An improvement value < 0 indicated that the patient's condition has deteriorated. Then, the larger the value was, the better the improvement in state. The action that maximized the total reward value was regarded as the optimal action. And that was the optimal intervention combination for the corresponding state.

Combined with the mathematical model, this study collected the model decision time on the first day, the 7th day, and the 14th day after admission; the included decision time did not exceed 147 days. In summary, the state, action, and rewards were assessed every 7 days.

Each state and action were converted into a digital combination, for example, a patient's state could be expressed as 200112 and 200111. The behaviour combination was 110, 101, and so on. Patients in the same state with the same treatment actions could produce different state outcomes. The possibility of transitioning to different states represents the probability of metastasis.

#### 2.2.2. MDP Simulation

Through the data transformation, we obtained the following: the state set S and the action set A, π←random strategy, Q (*s, a*)←random function, and Returns (*s, a*)←empty table. Initially, (a) S_0_∈S and *A*_0_∈A (s) are randomly selected. According to the strategy π, starting from S_0_ and *A*_0_, the state is terminated when the state does not occur for a long time or does not exist. In any event, after 500,000 games, the value function is very well approximated. So, the number of cycles set in this study was 500,000.

For each (*s, a*) that appears in the sequence, calculate *G*, *G* = Returns (*s, a*) =  ∑_*s*,*a*_^Terminal^reward(*s*, *a*) add *G* to the list of Returns (*s, a*), Q (*s, a*) =  1/*n*∑Returns(*s*, *a*) = average (Returns (*s, a*)). For each S update strategy, *π*(*s*) ← *avg*max*Q*(*s*, *a*)

#### 2.2.3. Principles and Basic Methods of Treatment

The institute of acupuncture and moxibustion is the key specialty of the State Administration of TCM. Since 2010, the clinical path management of stroke has been implemented. The treatment plan is implemented in accordance with the diagnosis and treatment plan promulgated by the State Administration of TCM, which ensures the feasibility of this study.

## 3. Results

### 3.1. Case Data

The information description of the patients at the time of admission is detailed in Table 1 because progress notes' records were incomplete, and there were missing 31 patients' partially data. 12 patients were only missing ataxia data, 8 patients were missing sensory disturbance data, and 2 patients were missing visual field defects data. 9 patients were missing ataxia and sensory disturbance data. All missing data were randomly filled by a computer.

### 3.2. Markov Decision Analysis Results

#### 3.2.1. State Data Set and Action Data Set

The state data set consisted of six states (*i*_1_–*i*_6_), and 835 patients had 236 state combinations through data conversion. The action data set consisted of three actions (*a*_1_–*a*_3_) and eight combinations (see Table 2 for details).

Each patient had a corresponding combination of behaviours at the decision-making moment.  Table 3 shows the quantity of patients and the main corresponding behavioural combinations in the real world.

#### 3.2.2. Rewards and Transition Probability

A total of 236 states were randomly selected for treatment with a combination of random behaviours to achieve the next state. The difference in neurological function between the two states is called the “reward”. The transition probability is the probability of transferring between states within a certain period of time. For patients with stroke at the convalescence stage in the same state, a combination of behaviours could be used to obtain multiple combinations of states. As shown in Table 4, combined with the reward and the probability of transition, the MDP recommended that patients in this state took the 111 regimen, and that was rehabilitation and Chinese herbal decoction combined with acupuncture treatment.

#### 3.2.3. Treatment Decision for Patients with Stroke at the Convalescence Stage

After the data extraction and transformation and the MDP, 835 patients obtained a total of 236 states, each of which corresponded to the optimal solution.

*(1) Decision-Making for the Patients with Different Ages, Related Diseases, and No Complications*. According to the results of the MDP, patients with disease history, no complications, and mild dysfunction, aged more than 66 years or between 46 and 65 years, had 7 optimal decisions. The former adopted the 001 treatment plan. In other words, the frequency of acupuncture treatment was the highest; the latter adopted the 100 treatment scheme, and the frequency of rehabilitation treatment was the highest; patients who were 18 to 45 years old had 4 types of optimal decisions, of which 001 was the highest frequency. When the neurological function was moderate to severe, the highest frequency was the rehabilitation, Chinese herbal decoction, and acupuncture combined treatment plan (see Table 5 for details).

*(2) Decision-Making for Patients ≥66 Years Old without Complications*. According to the results of the MDP, for patients who were ≥66 years without complications and with mild neurological deficits, there were a total of 4 optimal combinations, of which the 100 treatment scheme had the highest frequency. There were 7 optimal combinations for patients with a history of related diseases, of which the frequency of acupuncture treatment with the 001 treatment plan was the highest. When the neurological function was moderate to severe, the 111 regimen had the highest frequency, i.e., using a combination of rehabilitation, Chinese herbal decoction, and acupuncture treatment (see Table 6 for details).

*(3) Decision-Making for Patients with Phlegm-Blocking Type and Qi Deficiency and Blood Stasis Type*. According to the results of the MDP, there were 7 kinds of optimal combinations for patients with phlegm-blocking type. Among these combinations, the 111 combination had the highest frequency, which was rehabilitation, Chinese herbal decoction, and acupuncture combined therapy. For patients with qi deficiency and blood stasis type, when the degree of neurological deficit was mild, there were 8 optimal combinations. Among them, the 001 treatment plan had the highest frequency, which was the acupuncture treatment. When the degree of neurological deficit was moderate to severe, there were 8 best combination recommendations, of which treatment plan 111, which was rehabilitation, Chinese herbal decoction, and acupuncture combined therapy, had the highest frequency (see Table 7 for details). Because the cases of wind-fired disturbance type, yin-deficiency type, and phlegm-heat type patients were small, according to the MDP, we could not obtain the corresponding optimal combination.

## 4. Discussion

Western countries have taken the lead in proposing CER in the face of increasing medical expenditures and limited medical resources. Unlike traditional randomized controlled trials (RCTs), RCTs are used in the field of efficacy research with the goal of determining the effectiveness of a treatment and focusing on whether predetermined interventions produce the desired results [20]. As a form of empirical medicine, the clinical efficacy of TCM is fundamental to its existence and development. This is a scientific method used to evaluate the actual clinical effect between Chinese and Western medical treatments during the recovery period of stroke. This method is the key to improving and researching the clinical treatment level and clinical practice of TCM [21].

The “holistic concept” is one of the primary features of the theoretical system of TCM. In the course of treatment, the holistic view requires the doctor to be “patient-centred”, rather than “disease-centred”, and the whole must be combined with local, physical and mental, medical and patient [22]. The overall view coincides with the idea of CER's “patient-centred outcome study.” Another major feature of the theoretical system of TCM is “dialectical treatment”, which reflects that Chinese medicine treatment is a complex and dynamic process. The choice of outcome indicators is also diversified. This complex change is difficult to verify with RCT, but CER is used to explore the options, and the evaluation appears to be more in line with the essence of TCM and theoretical characteristics. Stroke is a complex disease, which evolves and transforms rapidly. Therefore, one doctor chooses a treatment rely on patient's condition.

The CER is conforming with the theoretical system of TCM and is a good way to the clinical research, which is a suitable method for excavating the clinical treatment methods and convenient for answering the question of which type of treatment is suitable for such groups [23]. Therefore, this study introduced CER into the efficacy study among treatment methods for stroke at the convalescence stage.

A total of 835 patients with stroke at the convalescence stage who were admitted to the Third Affiliated Hospital of Zhejiang Chinese Medical University from January 2012 to January 2017 were included in the study. The patients' data were collected from EHRs, in the other word, the data came from the real clinical world, and there were many confounding factors. At present, most of the observation data are processed by the method of propensity score that achieves the aim of “close to random allocation data” [24]. However, the treatments of patients at the convalescence stage are dynamic which bring great challenges and difficulties about the statistical analysis. So, this study introduced the MDP by establishing a mathematical model to achieve the research purpose. In medicine, the MDP is commonly used in survival analysis and health economic evaluation of diseases, such as tumors [25], unstable angina [26], and AIDS [27].

Most of the CER data come from various databases, and the popularity of EHRs provides strong support for the development of CER. Recording EHRs is the medical staff's work, and these contain the diagnosis and treatment process of the patients (including outpatients, inpatients, and medical examiners) [28]. EHRs include the completed and detailed clinical information which is generated and recorded during hospitalization. Under the strict management by the Zhejiang Provincial Health Department, the medical files are relatively sound and can reflect the real-world treatment status. EHRs can provide particular information on patient characteristics, such as race, medical history, family history, disease, and treatment changes. Rich data resources and accurate data contents can reduce the cost of CER and practical trials and, meanwhile, increase the research effectiveness [29].

Past research implies that rehabilitation treatment can help stroke at convalescence stage patients to exert their own potential, promote the recovery of patients' physiological functions, avoid complications or secondary disorders caused by reduced activities, and reduce the degree of disability of patients [30, 31]. Using Chinese herbal medicine to treat patients with stroke recovery has certain advantages. It can adjust the body's physical enginery, promote the recovery of neural function, and reduce the disability rate [32, 33]. Pan's team [34]has found that Huanyu Ditan Linao Decoction can effectively improve the clinical efficacy of patients with hypertensive cerebral haemorrhage during recovery and improve their exercise and living ability. Zhu's team [35] had found that Qingnao Yiyuan Decoction could effectively improve patients' neurological deficit scores and downregulate the expression levels of S100 B and NES. As a green therapy, acupuncture has an irreplaceable position in the treatment of stroke at the convalescence stage. A large number of studies [36–42] have confirmed that acupuncture has a satisfactory therapeutic effect on the stroke at the convalescence stage. At present, the recovery period of acupuncture treatment for stroke can be roughly divided into two categories, for the treatment of limb functional dysfunction and the treatment of nonlimb functional dysfunction. For limb functional dysfunction, Y Zheng's team [36] conducted a meta-analysis of acupuncture treatment of limb dysfunction after stroke, including 16 RCT papers and 1280 patients. The conclusion was that acupuncture could improve the patient's motor function and quality of daily life. Acupuncture also has a good effect on nonlimb functional dysfunction, such as oculomotor palsy after stroke [37], poststroke pseudobulbar palsy [38], and poststroke dysphagia [39–42]. In summary, acupuncture plays an important role in the treatment of stroke at the convalescence stage, and the guideline is also recommended as a level I evidence.

This study was based on EHRs and the innovative use of CER combined with mathematical models for data mining of patients with stroke at the convalescence stage. To some extent, this study has evaluated the real-world clinical data and suggested treatment for stroke at the convalescence stage, which made it possible for patients with various stroke recovery periods to choose the best treatment. Combined with the characteristics of TCM, MDP theory can continue to be used for certain common diseases to improve clinical efficacy and maximize the benefits for healthcare workers, patients, and other parties.

However, this study has several limitations. First, all of the data were taken from EHRs, and missing data were unavoidable. The results should be interpreted cautiously because of the possible bias caused by the replacement of missing data. In addition, due to too much variety, different components of rehabilitation therapy, Chinese herbal decoction, and acupuncture prescription were classified as one action. As a result, the effectiveness of different components of them is not comparable. This would limit the value and performance of the MDP planning for stroke treatment. Another limitation is that the key characteristics representing the patient states were based on the results of an expert panel meeting and previous study [43], so it might be missed some information about stroke patients. Finally, the research data were collected only from the Third Affiliated Hospital of Zhejiang Chinese Medical University, and area studies can be tried in the future.

## 5. Conclusion

In summary, the MDP makes it possible to study the effectiveness of various treatment methods in stroke patients who are at the convalescence stage. However, this study was an exploratory study and provided a different research method for the RWS. The results obtained by using the MDP need further research. Further exploratory studies using MDP theory in other areas in which complex interventions are common would be worthwhile.

## Figures and Tables

**Table 1:** General information of patients with stroke at the convalescence stage.

	*n* = 835	Male *n* = 501	Female *n* = 334
Age *n* (%)			
18–45 years	51 (6.11)	40 (7.98)	11 (3.29)
46–65 years	251 (30.06)	173 (34.53)	78 (23.35)
≥66 years	533 (63.83)	288 (57.49)	245 (73.35)

Disease history *n* (%)			
None	76 (9.10)	48 (9.58)	28 (8.38)
Have one	262 (31.38)	165 (32.93)	97 (29.04)
Have at least two	497 (59.52)	288 (57.49)	209 (62.57)

Complication *n* (%)			
None	614 (73.53)	372 (74.25)	242 (72.46)
Have at least one	221 (26.47)	129 (25.75)	92 (27.54)

Western medicine diagnosis *n* (%)			
Ischaemic stroke	637 (76.29)	378 (75.45)	259 (77.54)
Haemorrhagic stroke	198 (23.71)	123 (24.55)	75 (22.46)

Syndrome differentiation of TCM *n* (%)			
Wind-fired disturbance type	13 (1.56)	8 (1.60)	5 (1.50)
Phlegm-blocking type	498 (59.64)	315 (62.87)	183 (54.79)
Yin-deficiency type	64 (7.66)	37 (7.39)	27 (8.08)
Qi deficiency and blood stasis type	227 (27.19)	123 (24.55)	104 (31.14)
Phlegm-heat type	33 (3.95)	18 (3.59)	15 (4.49)

Levels of neurological functional impairment *n* (%)			
Level 1 (0–2)	89 (10.66)	59 (11.78)	30 (8.98)
Level 2 (3–5)	138 (16.53)	85 (16.97)	53 (15.87)
Level 3 (6–12)	398 (47.66)	233 (46.51)	165 (49.40)
Level 4 (13–19)	171 (20.48)	104 (20.76)	67 (20.06)
Level 5 (20–29)	8 (0.96)	4 (0.80)	4 (1.20)
Incomplete data	31 (3.71)	16 (3.19)	15 (4.49)

**Table 2:** Action data set.

Code	*a *_1_	*a *_2_	*a *_3_
1	1	1	1
2	1	1	0
3	1	0	1
4	1	0	0
5	0	1	1
6	0	1	0
7	0	0	1
8	0	0	0

**Table 3:** Quantity of patients and the main corresponding behavioural combinations in the real world.

Patient's quantity	State						Corresponding behavioural combinations		
	*i*_1_	*i*_2_	*i*_3_	*i*_4_	*i*_5_	*i*_6_	*a*_1_	*a*_2_	*a*_3_
34	3	2	0	1	2	3	0	0	0
							0	0	1
							1	1	1
							0	1	1
							1	0	1
							1	1	0

25	2	2	0	1	2	3	1	1	1
							0	0	1
							0	0	0
							1	0	1
							0	1	0
							0	1	1
							1	0	0
							1	1	0
…	…	…	…	…	…		…	…	…

**Table 4:** Behavioural combinations between different states, corresponding rewards, and transition probabilities.

Code	Pretreatment state	Action	Posttreatment state	Reward	Transition probabilities
71	120223	111	120222	3	0.5151
		111	120223	1	0.0303
		001	120223	0	0.3333
		101	120223	0	0.1212

**Table 5:** Decision-making for patients with various ages, related diseases, and no complications. Decision-making for patients ≥66 years old without complications.

Degree of neurological deficit	≥66 years		46–65 years		18–45 years	
	Action	Frequency	Action	Frequency	Action	Frequency
Mild	000	4	000	1	001	3
	001	8	010	2	100	2
	010	4	100	6	101	2
	110	1	110	1	111	2
	101	2	101	1		
	011	1	011	3		
	111	2	111	4		

Moderate to severe	000	1	000	3	010	1
	001	1	001	2	110	1
	010	2	100	3	101	2
	100	1	110	1	011	2
	101	8	101	2	111	5
	011	5	111	15		
	111	9				

**Table 6:** Decision-making for patients ≥66 years old with no complications.

Degree of neurological deficit	Without disease history		With disease history	
	Action	Frequency	Action	Frequency
Mild	000	1	000	4
	001	1	001	8
	100	3	010	4
	110	1	110	1
			101	2
			011	1
			111	2

Moderate to severe	100	1	000	1
	101	1	001	1
	011	2	010	2
	111	3	100	1
			101	8
			011	5
			111	9
			000	4

**Table 7:** Decision-making for patients with phlegm-blocking type and qi deficiency and blood stasis type.

Degree of neurological deficit	Phlegm-blocking type		Qi deficiency and blood stasis type	
	Action	Frequency	Action	Frequency
Mild	000	7	000	4
	001	9	001	6
	010	3	010	2
	100	9	100	3
	101	5	110	4
	011	1	101	2
	111	10	011	4
			111	3

Moderate to severe	000	5	000	6
	001	5	001	2
	010	3	010	4
	110	2	100	3
	101	13	110	4
	011	5	101	5
	111	20	011	4
			111	15

## Data Availability

The data sets used and/or analyzed during the current study are available from the corresponding author upon reasonable request.

## Abbreviations

EHRs: Electronic health records;
MDP: Markov decision process;
RWS: Real-world study;
CER: Comparative effectiveness research;
TCM: Traditional chinese medicine;
RCTs: Randomized controlled trials.

## Supplementary Materials

Supplementary Materials Appendix 1: state, action, and corresponding values. Additional file 2 ( Appendix 2): neurological functional impairment assessment for patients with stroke at the convalescence stage.
