# Supplementary material for: Comparative Effectiveness of Different Combinations of Treatment Interventions in Patients with Stroke at the Convalescence Stage Based on the Markov Decision Process
Source: Evid Based Complement Alternat Med. 2020 May 12;2020:8961341. doi: 10.1155/2020/8961341 (PMC7240799; doi:10.1155/2020/8961341)
Supplement: Supplementary Materials — Appendix 1: state, action, and corresponding values. Additional file 2 ( Appendix 2): neurological functional impairment assessment for patients with stroke at the convalescence stage. [file 8961341.f1.docx]

**Additional file 1: Appendix 1：State, Action and corresponding values**

**1. States and values.**

*i_1_* = Age (1=18 to 45 years；2 = 46 to 65 years；3 = more than 66 years)

*i_2_* = Any history of disease, such as diabetes, hypertension, coronary heart disease, abnormal blood liquid level, or auricular fibrillation; (0 = None, 1= at least one)

*i_3_* = Any complication, such as pulmonary infection, urinary tract infection or deep vein thrombosis; (0 = None, 1 = at least one);

*i_4_* = Western medicine diagnosis; (1= ischaemic stroke; 2 = haemorrhagic stroke)

*i_5_* = Syndrome differentiation of TCM (Pattern of TCM): (1 = wind-fired disturbance type; 2 = phlegm-blocking type; 3 = yin-deficiency type; 4 = qi deficiency and blood stasis type 5 = phlegm-heat type)

*i_6_* = Level of neurological functional impairment: Neurological function impairment scores were divided into five grades, as follows: 1(0-2), level 2(3-5), level3(6-12), level 4 (13-19), level 5 (20-29).

**2. Actions and values**

*a_1_* = Used rehabilitation therapy or not; (0 = unused; 1 = used);

*a_2_* = Used traditional Chinese medicine decoction or not; (0 = unused; 1 = used);

*a_3_* = Used acupuncture treatment or not; (0 = unused; 1 = used).

**Additional file 2: Appendix 2. Neurological Functional Impairment Assessment for patients with stroke at the convalescence stage.**

| Item | Rating Criteria |
| --- | --- |
| 1.Level of consciousness | 0=awake; 1=Sleepiness ; 2=lethargy; 3=coma |
| 2.Visual field defects | 0=normal; 2=defected |
| 3.Facial paralysis | 0=normal; 2=facial paralysis |
| 4.Muscular strength of left upper limb | 0=level 5; 1=level 4; 2=level 3; 3=level 2; 4=level 1; 4=level 0 |
| 5.Muscular strength of left lower limb | 0=level 5; 1=level 4; 2=level 3; 3=level 2; 4=level 1; 4=level 0 |
| 6.Muscular strength of right upper limb | 0=level 5; 1=level 4; 2=level 3; 3=level 2; 4=level 1; 4=level 0 |
| 7.Muscular strength of right lower limb | 0=level 5; 1=level 4; 2=level 3; 3=level 2; 4=level 1; 4=level 0 |
| 8.Aphasia | 0=normal; 2=aphasia |
| 9.Dysarthria | 0=normal; 1=dysarthria |
| 10.Sensory disturbance | 0=normal; 1=feels obstacle |
| 11.Ataxia | 0=normal; 2=abnormal |
| Total scores:0 to 29 points | |
